# Railway Point-Operating Machine Fault Detection Using Unlabeled Signaling Sensor Data

**DOI:** 10.3390/s20092692

**Published:** 2020-05-09

**Authors:** Pritesh Mistry, Phil Lane, Paul Allen

**Affiliations:** School of Computing and Engineering, University of Huddersfield, Queensgate, Huddersfield HD1 3DH, UK; p.lane@hud.ac.uk (P.L.); p.d.allen@hud.ac.uk (P.A.)

**Keywords:** condition monitoring, signal processing, fast Fourier transform, railway point-operating machines, turnout, fault detection, unlabeled data, smart sensors

## Abstract

In this study, we propose a methodology for the identification of potential fault occurrences of railway point-operating machines, using unlabeled signal sensor data. Data supplied by Network Rail, UK, is processed using a fast Fourier transform signal processing approach, coupled with the mean and max current levels to identify potential faults in point-operating machines. The method developed can dynamically adapt to the behavioral characteristics of individual point-operating machines, thereby providing bespoke condition monitoring capabilities in situ and in real time. The work described in this paper is not unique to railway point-operating machines, rather the data pre-processing and methodology is readily applicable to any motorized device fitted with current sensing capabilities. The novelty of our approach is that it does not require pre-labelled data with historical fault occurrences and therefore closely resembles problems of the real world, with application for smart city infrastructure. Lastly, we demonstrate the problems faced with handling such data and the capability of our methodology to dynamically adapt to diverse data presentations.

## 1. Introduction

Railway point-operating machines (POM) are electro-mechanical devices that operate turnouts, enabling a train to be directed from one track onto another via tapering rails, known as switch blades. POMs are located at various points along a railway line and remotely operated to divert a train to the normal (NR), or reverse (RN) direction [1]. The NR direction allows a train to pass straight through a turnout, while the RN direction directs the train along the alternate path.

POM failures are considered critical failures of a rail network system. Signaling equipment and turnout failures have been accounted for 55% of all railway infrastructure component failures [2], leading to delays, costly repairs, and potentially hazardous situations [3]. Where delays are not kept to within targets set out by regulators, substantial fines can be imposed onto the operator [3].

**Figure 1:**
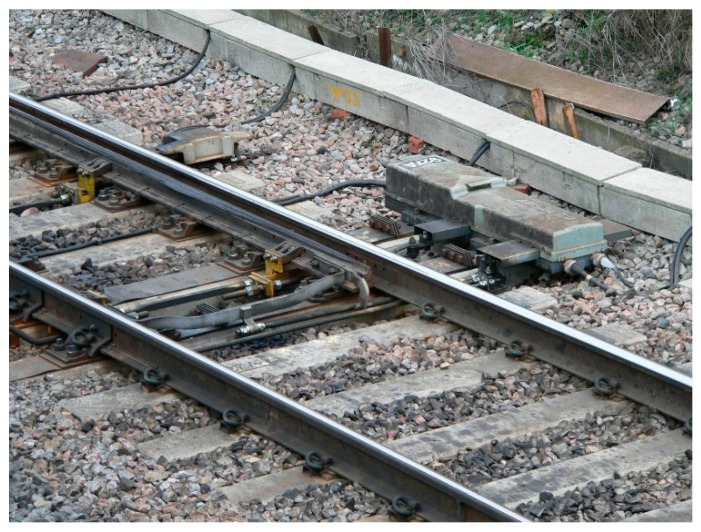
Point-operating machine (POM) (https://en.wikipedia.org/wiki/Railroad_switch).

A POM (Figure 1) will naturally undergo a process of degradation as operational wear takes hold, ultimately leading to complete failure. If progression to failure can be identified, preventative maintenance can be scheduled, avoiding operational down time and costly repairs. The currently practiced methods for POM maintenance include replacement after a fixed time period or beyond an accumulated number of actuation operations. However, they do not consider environmental influences such as track load or weather exposure [3]. A consequence of this may be that faults occur much sooner than expected or appear sporadically without explanation. More dynamic detection methods are thus sought to help monitor such occurrences.

Early fault detection methods relied solely on threshold settings [4], but produced limited success with a high false positive detection rate [5]. A thresholding method is unable to foresee a potential fault developing, unless the threshold is set unnecessarily lower than optimal. It also adopts a one-size-fits-all approach, and is not adaptable to the individual behavioral characteristics of mechanical devices [3].

Statistical approaches have fared better, [2,6,7,8,9,10] and provide the added advantage of being able to function in real time, without the need for historically labelled data of “good” and “bad” examples. Garcia et al., used the Kalman filter approach and compared new data to a reference point to identify faults. Their relatively straight forward approach was able to detect the majority of faults as they occurred [2]. Statistical feature extraction of signal data followed by clustering techniques have been successfully demonstrated as a useful fault detection approach. A study that extracted over ten statistical features from their signaling data, discovered that many of the faults investigated were identifiable by only two features; the maximum force and the root mean square (RMS) [7]. Evidently there are clear advantages for using a statistical approach, notably they are well understood endpoints, with relatively fast computations and therefore able to highlight attributes of the signal data that are not otherwise visible.

Recently reported work has covered the vast field of machine learning techniques, with labelled data to train intelligent models to predict future failures. Although highly successful, the downside of such models is the requirement of labelled data to train a model, which is not readily available in a real time setting. Nevertheless, we shall briefly discuss some machine learning approaches discovered from the literature used in condition monitoring applications.

For POMs producing signal data, the predominant condition monitoring approach seems to be that of support vector machines (SVM). SVM models are margin-based discriminant classifiers, shown to perform well with such data. For example, using discrete wavelet transform to extract features at different levels of decomposition, SVM classifiers built with these features were highly accurate in classifying faults, included being able to classify the severity of fault occurrence [11]. Vileiniskis et al., used features extracted from non-faulty POM actuations to develop decision boundaries of a one-class SVM. They then tested their model with unseen data to see if the actuation signals were abnormal, which fell outside of this boundary, and would be flagged as failures [3]. Bian et al., combined self-organizing maps (SOM) with SVM to classify degradation states of POMs, achieving accuracies in the region of 96 % [9]. Using principal component analysis for feature reduction, Eker et al., developed a SVM fault detection model that achieved 100 % accuracies [10]. It is worthwhile to note that studies reporting high accuracies were often found to be using small datasets or simulated data to build models. Although this is common practice in the domain, these models may perform comparatively when presented with larger volumes of real-world examples.

Shape analysis is also a common approach to condition monitoring problems, and more relevant to this study than the use of classifiers alone. Shape analysis primarily focuses on understanding the signal shape profiles of normal and abnormal actuation events, attempting to identify subtle differences caused by the aging of an instrument. These differences can then be modelled for fault identification. Sa et al., used a shapelet algorithm which extracts a subsequence of the time series called a shapelet. The shapelet attempts to minimize the in-class distance while maximizing the between-class distance of examples in the dataset. They used these extracted subsequences with a decision tree model, producing accuracies of 97 % [1]. In a similar study, Garcia et al., compared the expected shapes of signals and used a vector autoregressive moving-average model (VARMA) to forecast the outcome of new signals. If the new signal resulted in a signal beyond the expectation of the model a fault was considered to have been detected [12].

In this study, we use real-world unlabeled data collected from POMs to identify potential fault occurrences using a fast Fourier transform and curve fitting approach. It builds on our previously reported work in this area [13]. We corroborate our methodology against more traditional thresholding approaches to evaluate the effectiveness of our proposed method.

The remainder of this article is arranged as follows: Section 2 details the data used in this study and the pre-processing steps leading to actuation extraction. Section 3 presents the methodology of this work, more specifically, feature extract from actuation signal data, fast Fourier transform, and curve fitting, which are all integral parts of the proposed methodology. The results of this work with interpretation of findings are detailed in Section 4. Lastly the work is concluded in Section 5.

## 2. Signal Sensor Data

In this study, we use electrical current draw data collected by sensors attached to POMs across three regions of the United Kingdom (UK); London North-Eastern (LNE), London North-Western (LNWN) and Sussex. Data have been collected and supplied by Network Rail, UK, and contains the records of 331 POMs, whose actuations have been recorded over a period from April 2018 to June 2018.

The data for each region consists of several thousand csv (comma-separated values) files, each file containing a daily log of all the actuation operations performed by any particular POM in a given direction (NR or RN). Each file contains the current draw in amperes (amps or A) against a time stamp recorded in milliseconds (ms) as a continuous sequential log for any given day. Depending on the physical location of a particular POM, an instrument on a busy railway line will naturally contain many more actuations per day than a POM located on an infrequently used railway line. The daily log files which make up the dataset are therefore populated with a varying number of actuations according to frequency of POM use. 

Table 1 shows the number of files and POMs in each of the three regions initially available from the dataset. The data are considered to be unlabeled as no indication of fault occurrences within the logged events or otherwise are available.

An example of a subset of data extracted for point-operating machine POM-1 moving in the NR direction is shown in Table 2. These data were recorded on the 1st June 2018, with the initial data entry beginning at 02:28:05.527 in the morning. The table shows the first five records (Row0-Row4) and the last five records (Row242-Row246) of the first actuation. The first five records of the subsequent actuation are also shown (Row247-Row251). This example illustrates the continuous sequential way the data are recorded. The frequency of which the data are recorded during an actuation is approximately 10 ms. Once an actuation has executed, data recording stops until the next operation of the POM is actioned. Since the frequency of data records in any given actuation is approximately 10 ms, we can distinguish between two sequential actuations by noting the time difference between each record. Row246 and Row247 of Table 2 represent the end of one actuation (Row246) and the beginning of the next actuation (Row247) for point-operating machine POM-1 in the NR direction. The time stamp for these records are recorded as 2018-06-01 02:28:07.977 (Row246) and 2018-06-01 05:30:57.793 (Row247). This time difference equates to many minutes and indicates that those two records cannot belong to the same actuation event. Lastly, we can see from this data that for any given actuation, the current draw recording begins at a value of 0.00 A (zero amps) and ends with a recorded value of 0.00 A. This is seen for the records at Row0 (start of the first actuation) and Row246 (end of the first actuation), then again at Row247 (start of second actuation).

Table 2 represents a typical example of the data files contained within the larger dataset available for this study. The frequency of data records is 10 ms, although for some files, individual records that exceeded a 10 ms frequency is observed.

### 2.1. Pre-Processing

Where real-world data is concerned, it is seldom free of error. As such, a significant element of pre-processing was required. For this study, all our data pre-processing and analysis was performed using Knime analytics platform [14] and R Statistical package [15]. Where sensors are concerned, missing records are common errors due to sensor malfunctions and other equipment failures. To begin, all 45,690 files were checked for errors. Files that contained missing values or erroneous values which resulted in logged data not akin to that shown in Table 2 were removed from the dataset. Most of the errors identified involved data files which were entirely absent of any recorded data.

Although the csv files in our dataset were labelled with the operating point machine name and date of data collection, we discovered several csv files in different folders with identical file names. Unable to ascertain the validity of these files, all files with duplicate file names were also removed from the dataset. 

For sensor data of this nature or similar, many different types of errors can present themselves in the final dataset. As such, the data cleansing process is often unique to the task at hand. Since our dataset was large, removal of erroneous files was not an issue. For smaller datasets, an appropriate method for handling erroneous data would be more appropriate. The initial pre-processing step described, reduced the dataset from 45,690 files to 23,389 files, as shown in Table 3.

### 2.2. Actuation Extraction

Once the data was cleansed of errors, the remaining 23,389 files were further processed. The methodology proposed in this paper requires each individual actuation to be processed separately. This is required so that features can be extracted from each actuation. In this study, the direction of actuation, either NR or RN, were not treated separately. Rather the actuation for a given machine in either direction was simply treated as a sequential movement operation performed by the POM. For this initial study, the assumption was made that the occurrence of a fault would be apparent regardless of the direction of movement.

For our dataset, the frequency of current draw measurements was typically 10 ms between records as seen in Table 2. On occasion a value greater than 10 ms, generally between 20-100, is observed. Given these observations, it was decided that a timestamp difference of greater than 500 ms would be used as the threshold value for distinguishing between adjacent actuations in the sequential data records.

As an example, records in Row0-Row246 of Table 2 all show a time difference of 10 ms between subsequent records. However, the time difference between Row246 (2018-06-01 02:28:07.977) and Row247 (2018-06-01 05:30:57.793) of Table 2 is greater than 500 ms. This difference of greater than 500 ms indicates the end of one actuation and the start of the subsequent actuation. It is also worthy of mention that all actuation events begin with a current reading of 0.00 A (zero amps) and end with a current reading of 0.00 A. This can be seen in Table 2 for records Row0 (start of first actuation) and Row246 (end of first actuation), and once again for Row247, which is the start of the subsequent actuation.

**Figure 2:**
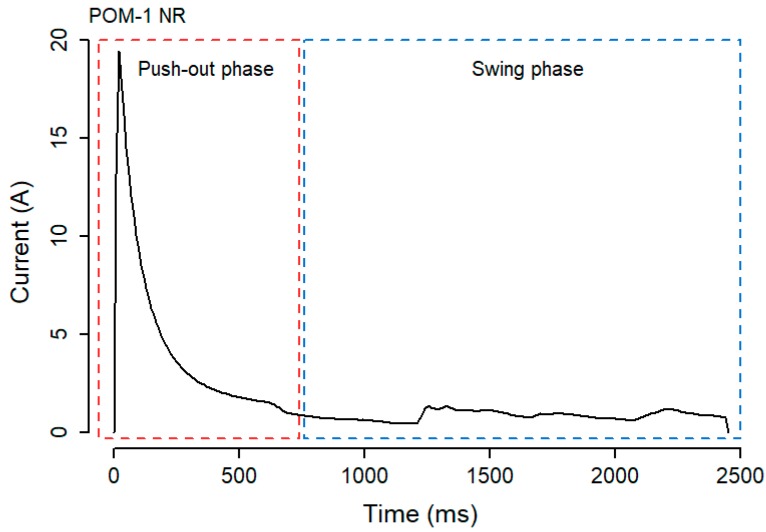
Current draw signal of a single actuation event for point-operating machine POM-1, moving in the NR direction, recorded on the 01st June 2018. The push-out phase (red) and swing phase (blue) of the actuation event are shown.

A typical current draw signal of a single actuation event for point-operating machine POM-1 is shown in Figure 2. This event was recorded on the 01st June 2018 with the instrument moving in the NR direction. Some of the data points for this actuation are shown in Table 2. In this paper, we describe two phases of an actuation event—the push-out phase and the swing phase. These phases are used in our proposed methodology to extract features from the actuation event. In other published work, Vileiniskis et al. [3], further defines the push-out phase to consist of two stages—the motor start-up and the unlocking stages. The swing phase also consists of two stages—the switch rail movement and the locking stages. Other studies also describe the actuation signal similarly [5,9,16]. For the purpose of this study, we will keep with the two phases shown in Figure 2 namely the push-out phase and the swing phase.

## 3. Method

After isolation of actuation signals for each POM, as described above, the fault detection methodology proposed in this study involves the extraction of three principal features from these signals. These features are then used to develop our fault detection capabilities. The three features extracted are as follows:

1. Extraction of the maximum current draw (peak current) and time duration of the push-out phase.
2. Extraction of the mean current draw and time duration of the swing phase.
3. Fast Fourier Transform analysis of the time domain signal into frequency domain representations, followed by goodness of fit analysis of a linear fitted cure.

**Figure 3:**
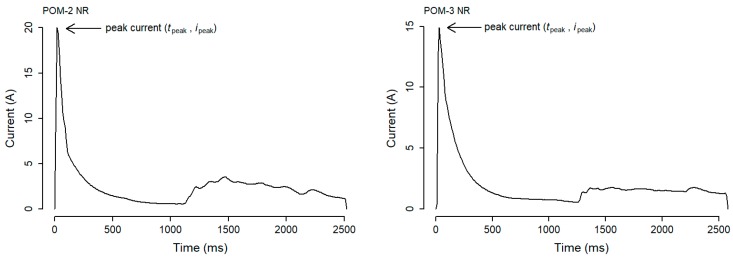
Current draw signal of actuation events for point-operating machines POM-2 (peak current ≈ 20A) and POM-3 (peak current ≈ 15A) in the NR direction.

To isolate the push-out phase from the swing phase for any given actuation, a fixed time duration could be used as a cut-off point to separate the two phases. However, the current draw signal produced by POMs are not only unique between different instruments but also change significantly for the same POM over time. Figure 3 shows the current draw signal of two different POMs moving in the NR direction. The signals produced are visibly different in several ways, for example, the maximum current value (peak current) is noticeably different for the two instruments. The signal produced by the two POMs after the 1000 ms point are also noticeably different.

For any single actuation event, the current draw signal is represented by two arrays, *I* and *T*, of equal size, n. Where *I* = [*i*_1_…*i*_n_] and *T* = [*t*_1_…*t*_n_], I represents the current draw array, while *T* denotes the time array. The start and end points of the push-out phase signal can therefore be denoted by (*t*_pstart_, *i*_pstart_) and (*t*_pend_, *i*_pend_) respectively, and similarly the start and end points of the swing phase signal can be denoted by (*t*_sstart_, *i*_sstart_) and (*t*_send_, *i*_send_) respectively.

To automate the separation of the push-out phase from the swing phase, we employed a gradient of the slope method, calculated using pair-wise sequential adjacent records. For current draw, *i*, and time, *t*, then;

(1) $$gradient\;\left(grad_{n}\right)=\frac{\left[i\right]-\left[i-1\right]}{\left[t\right]-\left[t-1\right]}$$

The aim of the gradient of the slope method is to identify point (*t*_sstart_, *i*_sstart_) in the signal data. For any given actuation, the signal begins with a positive gradient from point (*t*_pstart_, *i*_pstart_) up to the peak current point (denoted here as (*t*_peak_, *i*_peak_), see Figure 3). After the peak current, the gradient turns negative as the current draw value descends. At some point during this descend the gradient becomes non-negative (i.e., grad ≥ 0.00), which we deem to be the start of the swing phase. To identify the start of the swing phase (*t*_sstart_, *i*_sstart_), an array, *G*, of length n, is represented by

(2) $$G=\{\begin{array}{l} 1,\;\mathrm{if}\;(grad_{n}\geq 0\;\mathrm{and}\;grad_{n-1}<0)\;\\ 0,\;\mathrm{otherwise} \end{array}$$

The index position at which the first occurrence of “1” appears in array *G*, therefore, corresponds to the index position of array, *I*, and array, *T*, whose values correspond to the coordinates for (*t*_sstart_, *i*_sstart_). This therefore identifies the point along the actuation signal which corresponds to the beginning of the swing phase.

Upon separation of the push-out phase from the swing phase, the following features are extracted.

Peak current: The peak current (*t*_peak_, *i*_peak_) of a signal is found in the push-out phase of an actuation (see Figure 3). The peak current is taken as the maximum single current draw value found in the push-out phase.

Push-out phase time duration: The push-out duration is taken as the time difference in milliseconds (ms) between *t*_pend_ and *t*_pstart_.

Mean current of the swing phase: The mean current draw of the swing phase is taken as the arithmetic mean current value of the swing phase.

Swing phase time duration: The swing duration is taken as the time difference in milliseconds (ms) between *t*_send_ and *t*_sstart_.

### 3.1. Fast Fourier Transform Analysis

The time series signal data of an actuation event of a POM is shown in Figure 2. Fast Fourier transform (FFT) facilitates the analysis of such signals in the time domain, to deconstruct components into a frequency domain representation. It is then possible to analyze the different frequencies present in the original signal using the frequency domain components that are represented as discrete values or bins (discrete Fourier transform (DFT)). The frequency domain is useful at showing if noise or jitter is contained within your time domain signal, which is helpful when attempting to identify spurious actuation signals from unlabeled data. The principles of FFT are well explained in the literature [17,18] and so not explained further. The details of the application of FFT for this study is discussed herein.

**Figure 4:**
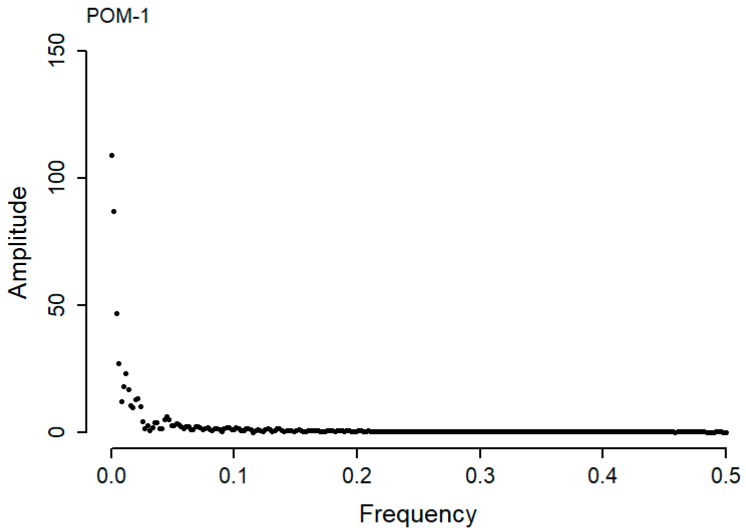
Fast Fourier transform, frequency domain plot of the time domain signal data shown in Table 2 for point-operating machine POM-1, moving in the NR direction.

Typically, each single actuation event in the dataset resulted in approximately 250 sample points. To ensure all actuation events were processed equally, a zero padding was added to the end of every actuation signal which resulted in all profiles containing 512 samples and allowing quicker computation time. A sliding window of size 512, together with a step size of 256 was used with the input signal. The Hann windowing function was applied to the signal, this minimizes spectral leakage, allowing the FFT to extract spectral data, and prevents it from producing the wrong frequencies. FFT assumes a finite dataset, which can be thought of as a single period of a periodic signal. If data are not of periodic circular topologies, windowing using the Hann function, reduces the amplitude of the discontinuous data at the boundaries of the signal. A FFT of the signal domain data shown in Table 2, for point-operating machine POM-1 moving in the NR direction, is plotted as a frequency domain plot in Figure 4.

### 3.2. Curve Fitting

For actuation events to be compared, it is necessary to process the frequency domain plots produced (see Figure 4). Anomalies in actuation events can then be identified to indicate possible fault occurrence in the POM. An attempt to fit second and third order polynomials to the curve proved unsuccessful, although other studies have been more successful with their data [16]. Thus, the frequency domain signal was further processed by taking the log_10_ values of both the Amplitude and the Frequency values produced from the FFT analysis.

**Figure 5:**
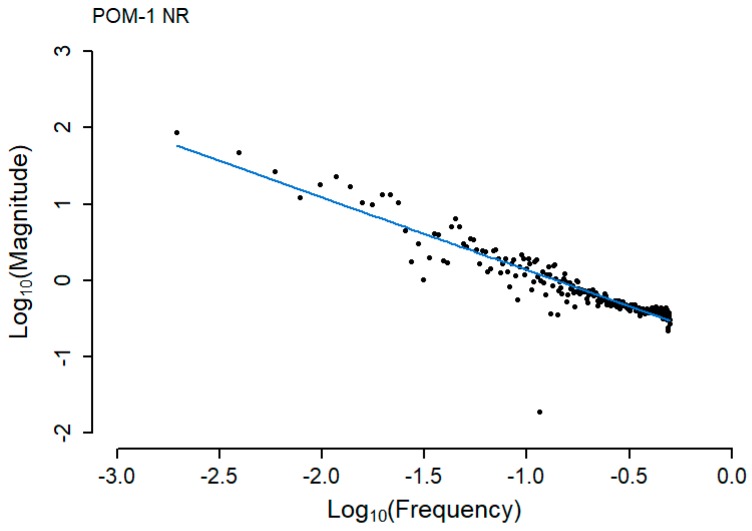
Linear curve fitted to the log_10_ transformations of the FFT analysis outputs for point-operating machine POM-1 in the NR direction.

Log_10_ calculations produce a more linear relationship between the Amplitude and Frequency domain, allowing a linear curve to be fitted and the goodness of fit calculated. The frequency domain data used in Figure 4 has been log_10_ transformed and fitted with a linear curve using the R statistical package [15]. A plot of this data is shown in Figure 5 below.

After curve fitting, the goodness of fit is assessed as the residual standard error (RSE). This makes for the final feature extracted for our fault detection analysis.

These observed residuals are subsequently used as an estimation parameter of the variability in the samples. When the RSE is 0 (zero), the curve fits the data perfectly, in the real world, this would likely be due to overfitting. The RSE in this study becomes useful as a measure of variability in the data, which can imply current draw variability in the actuation event of a POM. Thus, a high RSE value for one actuation event may indicate fault occurrence when compared to an actuation with a lower RSE value. In this study, we used an arbitrary RSE threshold value of ≥0.3 to serve as a guide to indicate error in the actuation signal. In practice this value would need to be set dynamically depending on the baseline value of each individual POM. For this study, a value of ≥0.3 was used across all instruments.

## 4. Results

In this section, we discuss the outcomes of the methodology described above, which is based on the proposition that an abnormally functioning POM, can be identified from the current draw signal data that it produces. Identifying irregular patterns, are indicative of likely signs of wear or imminent failure [1]. The data used in this study are unlabeled data, here we compare actuation events from POMs that have been processed by the method described and show how potential fault occurrences can be identified. These candidates would then be flagged up for review and investigation.

Our feature extraction approach used traditional methods of mean current and max current draw that have been used and widely reported in other studies [16]. Furthermore, we undertook a more specific FFT approach to extract frequency domain features which were transformed into the linear form before calculating the RSE of the curve fitted. The RSE is used as an additional feature as part of our analysis.

**Figure 6:**
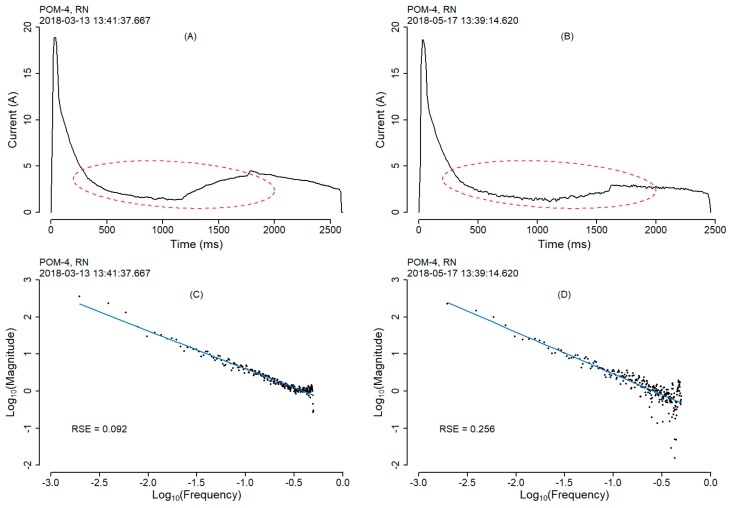
Actuation events of point-operating machine POM-4 in the RN direction. The events are recorded on two separate days—2018-03-13 (**A**) and 2018-05-17 (**B**)—with their FFT log_10_ transformation and linear curve from each event directly below (**C**,**D**). The red ellipse (**A**,**B**) highlights the area of the two actuation events that show variability in the signal, which is reflected in the observed RSE values.

**Figure 7:**
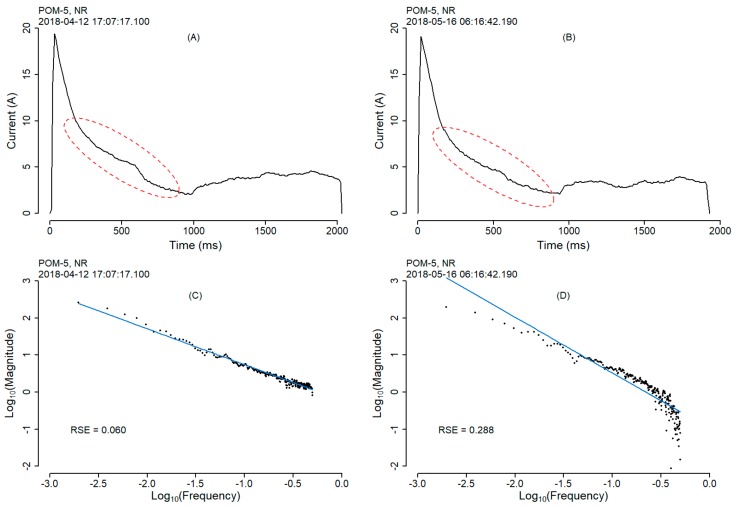
Actuation events of point-operating machine POM-5 in the NR direction. The events are recorded on two separate days—2018-04-12 (**A**) and 2018-05-16 (**B**)—with their FFT log_10_ transformation and linear curve from each event directly below (**C**,**D**). The red ellipse (**A**,**B**) highlights the area of the two actuation events that show variability in the signal, which is reflected in the observed RSE values.

To demonstrate the FFT approach, two examples of two different POMs are shown below. Figure 6 and Figure 7, show the current draw signal of point-operating machine POM-4 and POM-5 respectively.

Figure 6 shows two actuation events for point-operating machine POM-4 moving in the RN direction recorded on the 2018-03-13 and 2018-05-17 (Figure 6A,B). Following the method proposed above, the RSE values calculated from these two actuation events following FFT log_10_ transformation and linear curve fitting, suggest that the actuation produced at the later date (Figure 6) is indicative of potential failure or error. We can see from the highlighted areas (red ellipse) where the variability in the signal occurs between the two individual events. Furthermore, the potential error signal (Figure 6B) produces a higher RSE value of 0.256 (Figure 6C) compared to the earlier actuation, which produces a RSE value of 0.092 (Figure 6D). The fitted curve of Figure 6C shows how the FFT log_10_ transformation of the frequency and magnitude domain data points, fit much more closely to the curve resulting in a much lower RSE value than that of Figure 6D.

Likewise, in Figure 7 we present the actuation events for point-operating machine POM-5 moving in the NR direction, with the events recorded on two separate dates. Once again, we show the variability in the actuation signals (Figure 7A,B) and the subsequent curve fitting following FFT log_10_ transformation (Figure 7C,D). The actuation event which results in a greater degree of variability produces a higher RSE value of 0.288 (Figure 7D), compared to the event from an earlier date, which produced a RSE value of 0.060 (Figure 7C).

Similar observations are made throughout the dataset for other POMs where potential faults can be flagged using the method proposed. Interestingly, the signal profiles of point-operating machine POM-4 (Figure 6) are very different when compared visually to that of point-operating machine POM-5 (Figure 7). The areas of the actuation signal where variability is found is clearly not fixed between different POMs. We also observe that the max peak current for point-operating machine POM-4 for both the actuation events (Figure 6A,B) is approximately 19 A. The same is observed for point-operating machine POM-5 (Figure 7A,B). Therefore, we cannot exclusively rely on single events such as max peak current as error indicators. Thus, the method proposed in this study allows the entire signal to be processed accounting for instrument variation.

**Figure 8:**
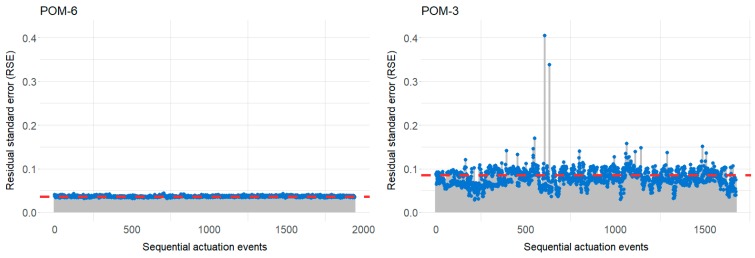
Sequential RSE values plots for point-operating machine POM-6 (*n* = 1943) and POM-3 (*n* = 1674). Possible baseline for each instrument shown in red.

For each instrument in the dataset, we have processed the entirety of the actuation events that have been recorded. It is, therefore, possible to observe the RSE values for each of these events to produce a characteristic profile for a given machine. Plotting the RSE against time, for each instrument quickly identifies actuations in the instrument’s timeline where it has potentially failed or about to fail. It also helps identify the baseline RSE value for any given instrument. To illustrate this point, two contrasting examples have been selected and are shown in Figure 8 below. It shows the calculated RSE values for point-operating machines POM-6 and POM-3 plotted sequentially. From these plots, it can be readily observed that point-operating machine POM-6, produced RSE values that are very consistent to one another (1943 actuations). This RSE value, of approximately 0.04, seems to be the baseline value (red line) for this machine. In contrast, point-operating machine POM-3, produced RSE values that were much less consistent (1674 actuations), but nevertheless a baseline value of approximately 0.90 could be inferred.

This again demonstrates that different POMs will all have their individual characteristics and therefore a methodology that can be adaptive is more suited to the type of challenge addressed in this study.

Interestingly, point-operating machine POM-3, resulted in two RSE values of ≥0.3, which is the threshold value used in this study to indicate potential fault via the methodology proposed.

In line with the more conventional approaches of signal fault detection, two additional useful features were extracted from our actuation signal data; 1) The maximum current draw (peak current) of the push-out phase and 2) The mean current draw of the swing phase. We can use these features to validate our methodology proposed in this study. Plotting the mean current, the peak current and the RSE on a scatter plot produces some interesting trends between different POMs. 

To illustrate this, two examples have been selected on the basis that their processed actuation events resulted in at least one RSE value of ≥0.3. We selected such instruments so that a plot of the peak current (push-out phase) against the mean current (swing phase) of the actuation events could be differentiated by RSE. 

**Figure 9:**
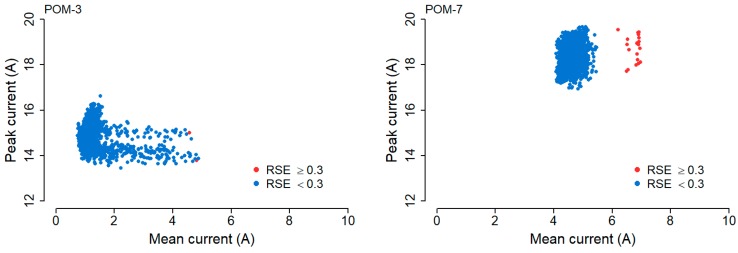
Mean current against Peak current of actuation signal data for point-operating machine POM-3 and POM-7. Data points of RSE values ≥0.3 (red), <0.3 (blue).

Figure 9 shows a scatter chart for point-operating machine POM-3 and POM-7. The peak current (A) of the push-out phase is plotted against the mean current (A) of the swing phase, with the data points colored (red or blue) according to their RSE value. Again, we see that the pattern of the scatter plots for the two POMs are quite different. Point-operating machine POM-7 contains data that is much more tightly clustered together and generally of higher peak current and mean current values than that of point-operating machine POM-3. What we do see in common however is the locations of the data points of RSE value ≥0.3. For both point-operating instruments, the data points which meet the threshold of RSE ≥0.3 are pushed out to the right of the major cluster.

## 5. Conclusions

In this study, we have proposed a methodology for the identification of potential fault occurrences from current and time signal data of POMs. Data acquired from Network Rail, UK, was cleansed and pre-processed, with subsequent work focusing on the exploration of a potential condition monitoring methodology suitable for motorized devices fitted with current sensing capabilities.

The developed method combines mean and max current level thresholds for the push-out and swing phases with an FFT-based approach which is dynamically suited to the individualistic nature of different POMs. Many studies have been reported in the literature that process labelled data, using a multitude of approaches, to develop condition monitoring capabilities. The novelty in the method we propose is that it uses a large volume of real-world unlabeled data. As such we are not pre-aware of fault occurrences within the data.

It is apparent from our efforts that observable differences are evident from the actuation signals of perceived good and poorly functioning POMs. We exploit these behavioral differences to develop fault sensing capabilities to flag instruments where malfunction could be imminent. This work highlights the uniqueness of mechanized motorized devices, and the requirement to move away from a one-size-fits-all approach. Our methodology can consider each instrument on a case by case basis and therefore monitor at the individual level. The method is unaffected by uncontrollable factors such as environmental conditions or varying operating characteristics which will naturally vary from machine to machine.

A limitation not addressed in this study was the identification of unique RSE threshold values, whereby an initial value of ≥0.3, was selected based on the data sets being studied in the earlier stages of the work. However, as our latter results demonstrate (see Figure 8), some instruments may require much lower thresholds, with the potential to overlook faults if this is not considered. In future work we will look at addressing this dynamic thresholding issue by using some form of machine learning approach. It may be more appropriate to use a clustering technique to identify “outliers” in the scatter plots of peak current against mean current, such techniques would accommodate for the unique behavioral characteristics of each instrument. In the field we envisage this machine learning to be realized by grouping machines of the same type and using the analytics engine to establish a threshold value relative to the performance of the machines in a particular group.

It is also worthy of note that the direction of the point movement, regarding it being either the out-swing (NR) or the return (RN) motion was not considered separately in this study. It would be worthwhile separating the actuations of the two directional movements to see if this produces more conclusive results. In other reported studies, a difference with respect to directional movement have been reported [2]. Unfortunately, although timestamps were an integral part of our data records, we could not use this feature more effectively since we did not know when, in the history of a POM, where failures or repairs had taken place.

In summary, this work has demonstrated a feasible approach to identify potentially failing POMs, using data processing techniques that could readily be integrated into a wider remote condition monitoring architecture.

## Figures and Tables

**Table 1:** Files present in the dataset for each region.

Region	No. of Files	No. of POMs
LNE	15,802	103
LNWN	17,081	129
Sussex	12,807	99

**Table 2:** Current (A) draw signal data for point-operating machine POM-1 moving in the NR direction.

Row No.	Date Time	POM-1 NR Current
Row0	2018-06-01 02:28:05.527	0.00
Row1	2018-06-01 02:28:05.537	13.18
Row2	2018-06-01 02:28:05.547	19.43
Row3	2018-06-01 02:28:05.557	18.04
Row4	2018-06-01 02:28:05.567	16.15
⋮	⋮	⋮
Row242	2018-06-01 02:28:07.937	0.82
Row243	2018-06-01 02:28:07.947	0.81
Row244	2018-06-01 02:28:07.957	0.80
Row245	2018-06-01 02:28:07.967	0.76
Row246	2018-06-01 02:28:07.977	0.00
Row247	2018-06-01 05:30:57.793	0.00
Row248	2018-06-01 05:30:57.803	0.81
Row249	2018-06-01 05:30:57.813	16.22
Row250	2018-06-01 05:30:57.823	19.37
Row251	2018-06-01 05:30:57.833	17.55
⋮	⋮	⋮

**Table 3:** Remaining files after removal or erroneous files.

Region	No. of Files	No. of POMs
LNE	6533	97
LNWN	9715	105
Sussex	7141	91
